# What is Susac syndrome? - A brief review of articles

**Published:** 2014-10-06

**Authors:** Ferdos Nazari, Amirreza Azimi, Siamak Abdi

**Affiliations:** 1 Department of Neurology, School of Medicine, Shariati Hospital, Tehran University of Medical Sciences, Tehran, Iran; 2 Department of Neurology, School of Medicine, Sina Hospital, Tehran University of Medical Sciences, Tehran, Iran

**Keywords:** Susac Syndrome, Branch Retinal Artery Occlusion, Sensorineural Hearing Loss

## Abstract

Susac’s syndrome (SS) is a clinical triad of encephalopathy, branch retinal artery occlusion and sensorineural hearing loss and maybe due to an immune-mediated endotheliopathy. Because of its rarity and some similarities to other common neurological conditions such as multiple sclerosis and acute disseminated encephalomyelitis, it is often misdiagnosed and therefore mistreated. To the best of our knowledge, there is only one case report from our country with this diagnosis. Here, we have a short discussion on this issue to introduce it to our colleagues and remind it as a differential diagnosis in patients with unexplained encephalopathy.

## Introduction

Susac’s syndrome (SS) is a clinical triad of encephalopathy, branch retinal artery occlusion (BRAO) and sensory neural hearing loss (SNHL), first described by Susac.^1^ He first reported two cases of brain and retinal vasculopathy in female patients with good response to corticosteroids in 1979.^2^ It is a rare syndrome and, until now, slightly more than 300 cases have been reported in the literature.^3^ SS is frequently seen in females of 20-40 years of age.^4^^,^^5^ It usually presents with severe headache and behavioral changes, progressive cognitive decline, apathy and later by hearing loss, tinnitus and segmental visual loss.^4^ The clinical triad of subacute encephalopathy, BRAO, and hearing loss is due to a precapillary arteriolar angiopathy of unknown origin, but most evidences are in favor of an immune-mediated endotheliopathy, and immunosuppressive therapy is the main mode of treatment.^6^^,^^7^

**Epidemiology**

SS is frequently seen in women with female to male ratio of 3:1, it’s onset varies in age of 9-58 years, but most of the patients are 20-40 years old.^4^^,^^5^ Mateen et al. in a series of 29 patients with SS reported that 83% of their patients were female with a mean age at symptomatic presentation of 35 (19-65) years.^8^ The overall incidence of SS is unknown, and there are limited case reports (more than 300 cases) of this syndrome in the literature.^3^^,^^5^

**Clinical Presentations**

Usually, there is no complete clinical triad at presentation.^4^ The encephalopathic syndrome may be heralded by severe and sometimes migrainous headache.^9^^-^^11^ Encephalopathy begins with behavioral abnormalities such as aggressiveness, paranoia, depression; then memory loss with confabulation, visuospatial deficits, diminished attention and concentration, disorientation, lethargy, and apathy may occur. This abnormal mental status sometimes wax and wane. During this period, urinary incontinence and generalized seizure may happen.^4^^,^^12^^-^^15^

In many of the reported cases of SS, sudden SNHL in association with peripheral vertigo, nystagmus and tinnitus occur after the encephalopathic features.^10^^,^^13^^,^^16^^,^^17^ The low and medium frequency SNHL occurs as a complication of cochlear apex arteriolar microinfarctions, may be permanent, and if severe, needs cochlear implantation.^18^

The third common clinical presentation of SS is BRAO, which means occlusion of some branches of the retinal artery due to endothelial injury. BRAO can lead to bilateral vision loss or remain asymptomatic, depending on the location of retinal involvement. Involvement of the posterior pole of the retina leads to profound blurred vision, but peripheral involvement can cause no symptom.^4^^,^^18^ BRAO also can cause photopsia, black spots, and scintillating scotomas.^10^ Therefore, detailed fundoscopic examination is necessary if there is any suspicion to SS. Ischemic retinal whitening resulting from BRAO is the most common finding on fundoscopy.^1^ Other findings are cotton-wool spots, tiny peripheral hemorrhages, edematous retina and Gass plaques.^13^^,^^15^^,^^19^^-^^21^

Gass plaques first described by Don Gass in patients with idiopathic BRAO, are yellow-white lipid deposits at the mid-segment of the retinal arterioles. These are caused by slow extravasation of blood lipids into the arteriolar wall at the site of arteriolar wall damage. Gass plaques are typically located at the mid-segment of the retinal arterioles and not at the arteriolar bifurcations, and usually are distant from retinal infarction.^19^ Gass plaques may be a transient finding and disappear during the remitting course of the illness.^10^

Skin involvement is not a common finding in SS. Turc et al. reported a 24-year-old man with a complete clinical triad of SS, who had skin lesions of livedo racemosa on his torso and feet. The lesions had not changed with warmth or cold, and had pathologic changes similar to the pathology of the brain biopsies of SS, reported earlier.^20^ They concluded that SS is a multi-organ disease and may involve other organs as well. Due to the asymptomatic nature of these skin lesions, Turc et al. have suggested a careful examination of the skin in patients suspicious to SS.^20^

**Pathogenesis**

The clinical triad of subacute encephalopathy, BRAO, and SNHL in SS is due to a microangiopathy of the precapillary arterioles of the brain, retina and inner ear.^4^ Most experts believe that this microangiopathy is due to an immune-mediated endotheliopthy that causes small vessel narrowing and occlusion, leading to microinfarctions of the brain (both white and grey matter), retina and cochlea. The endothelial barriers of the retina and cochlea are analogue to that of blood brain barrier, and these similarities can explain predilection of brain, retinal, and cochlear involvement in SS.^6^^,^^7^^,^^18^ Presence of anti-endothelial cell antibodies in some case reports is in favor of this immunologic pathophysiology, but it is not clear that these antibodies are the cause, or are produced secondarily. However, these antibodies are positive in other autoimmune disorders such as dermatomyositis and Sjogren syndrome.^10^

Another mean of understanding the pathophysiology of SS is pathologic study of biopsied specimens from these patients. The pathologic findings of brain biopsies from most of the case reports showed multiple microinfarctions of the brainstem, both grey and white matter, with the loss of axons, neurons and myelin in the lesions. Other common findings are swollen endothelial cells, endothelial proliferation of precapillary arterioles with marked thickening of the vessel walls, and minimal non-specific periarteriolar inflammatory cell infiltration.^8^^,^^10^^,^^14^^,^^15^^,^^18^ Similar pathologic features of microvascular thrombi and mild inflammatory cell infiltration have been observed in skin and muscle biopsies from some patients with SS, suggestive of a more diffuse disease than currently is known. Absence of necrosis in tissue biopsies suggests a microangiopathy, rather than vasculitis, as the leading cause.^9^^,^^18^ Some experts believe that the endothelial damage of the precapillary arterioles with deposition of the complement fragments begins an autoimmune anti-endothelial cell antibody syndrome, such as dermatomyositis, and as will be discussed later, consider similar treatment for SS and dermatomyositis.^11^

**Diagnosis**

SS is a rare disorder and, since a complete clinical triad is usually not present at first presentation,^4^^,^^22^ it can be easily misdiagnosed with other common clinical syndromes of encephalopathy (without BRAO and SNHL), especially acute disseminated encephalomyelitis (ADEM) or multiple sclerosis (MS). Because of some similarities between these conditions on magnetic resonance imaging (MRI), a false diagnosis can be made easily; also, improvement of the encephalopathy in response to a short course of steroid therapy reinforces the misdiagnosis. It’s only after recurrence of the illness during steroid tapering, and the emergence of other clinical features of SS, which an accurate diagnosis can be made.^17^ Many experts suggest consultation with a neuro-ophthalmologist or retinal specialist for detecting Gass plaques, in all patients with unexplained encephalopathy, to prevent misdiagnosis.^19^ There is no specific test for diagnosis of SS and the diagnosis should be made by characteristic clinical triad and exclusion of other conditions.^10^^,^^15^

There are different diagnostic tools for detecting brain, retina and inner ear abnormalities that help us for diagnosis of SS and also ruling out of other clinical disorders. Here, we are briefly pointing these diagnostic tools.

**MRI**

One of the major presentations of SS is brain involvement, and MRI is the neuroimaging tool of choice in this syndrome.^9^

**Figure 1:**
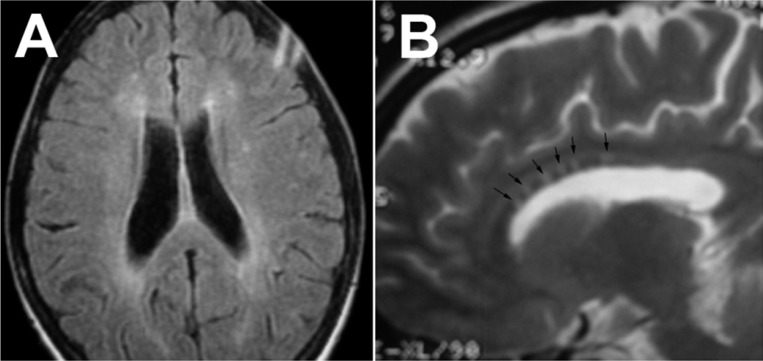
Periventricular and deep white matter involvement of the brain in Susac syndrome (A). Sagittal T2 showing multiple large central callosal “snowballs” (B) (courtesy of Dr. Sahraian MA)

As mentioned earlier, one of the most common presentations of SS is microinfarctions in the brain that involves both white and grey matter.^15^ These microinfarctions can cause T2 hyperintense lesions at any area of the brain, including periventricular and juxtacortical areas, subcortical white matter (Figure 1A), cerebellum and corpus callosum;^15^^,^^16^ but microinfarctions in SS can also produce two specific findings on MRI: “snowball lesions” that represent microinfarctions of the central part of the corpus callosum, best seen on sagittal T2 and Fluid-attenuated inversion recovery (FLAIR) views (Figure 1B); and “string of pearls” that represents micro-infarctions of the internal capsule, best seen on diffusion weighted image sequences.^17^^,^^18^ Rennebohm et al. believe that the combination of typical central callosal lesions with string of pearls is unequivocally pathognomonic for SS.^17^ Corpus callosum involvement is always seen in encephalopathic form of SS; snowball configuration of corpus callosum lesions in acute phase of encephalopathy ultimately evolves to central callosal holes, best seen on sagittal T1 sequence views.^17^ In post-encephalopathic stage, MRI shows these near-pathognomonic central callosal holes and linear defects in corpus callosum called “smokes.”^18^^,^^23^ Another MRI characteristics of SS is leptomeningeal enhancement, seen in 30% of patients during the acute phase; in some case reports, facial and vestibule-cochlear nerve enhancement and cochlear enhancement have been reported.^8^^,^^18^^,^^24^ Egan et al. believe that the imaging triad of the corpus callosum involvement, deep grey matter lesions and leptomeningeal enhancement, in an encephalopathic patient may help in early diagnosis of SS and warrant early, aggressive, treatment.^19^ After the acute phase, generalized atrophy of the cerebrum, cerebellum and corpus callosum can be seen in severely affected individuals.^10^^,^^11^ Mateen et al. reported a series of 29 cases of SS with a corpus callosum involvement in 79%, punctate T2-weighted hyperintense lesions in 93%, and gadolinium enhancing lesions in 50%, of their patients.^8^

The most common differential diagnoses of SS are MS and ADEM, but there are some distinguishing features as follows: (1) Corpus callosum involvement in SS is typically in the central portion of the corpus callosum and the periphery is spared, but in MS and ADEM the undersurface and septal interface of corpus callosum are involved. (2) Leptomeningeal enhancement is an uncommon finding in MS and ADEM but can be seen in up to 30% of patients with SS. (3) Involvement of deep grey matter including basal ganglia is a common finding in SS but unusual in MS.^10^^,^^18^^,^^24^

**Fluorescein angiography of the retina**

One of useful diagnostic test for detecting retinal artery involvement in SS is fluorescein angiography of the retina. Fluorescein angiography can show BRAOs as focal non-perfused retinal arterioles.^20^ Furthermore arteriolar wall hyper-fluorescence, dye leakage, retinal hemorrhage and edema may be seen. Arteriolar wall hyper-fluorescence means staining of the retinal arteriolar wall branches. It may occur in areas remote from the occluded arterioles. Presence of arteriolar wall hyper-fluorescence on fluorescein angiography in a patient with unexplained encephalopathy, even in the absence of BRAO, is highly suggestive of SS.^4^^,^^9^^,^^17^^,^^19^ As Mallan et al. have mentioned, arteriolar wall hyper-fluorescence is a marker of disease activity; serial fluorescein angiography can be used as a monitoring tool for assessment of response to immunosuppressive therapy. The presence of arteriolar wall hyper-fluorescence on fluorescein angiography without any visual complaint means subclinical disease activity and modifies our immunosuppressive treatment for minimizing visual impairment.^21^

**Other diagnostic tools**

***Lumbar puncture***

One of the most popular tests in patients with encephalopathy is the lumbar puncture; it is useful for ruling out many infectious causes of encephalopathy. In SS, there is usually a mild lymphocytic pleocytosis and mild elevation of cerebrospinal fluid protein. Oligoclonal bands are positive in some case reports and negative in others.^4^^,^^12^^-^^15^^,^^18^^,^^21^^,^^22^^,^^25^

**EEG**

In SS may show a non-specific diffuse slowing pattern, without epileptiform discharge.^4^^,^^12^ EEG may also show multifocal cerebral dysfunction with intermittent bursts of irregular slow waves over both hemispheres, without epileptiform discharges.^21^

**Perimetry**

In SS with retinal arteriolar involvement may show segmental visual field defects on the basis of involved arterioles.^4^^,^^21^^,^^25^

**Autoantibodies**

The most popular pathophysiology of SS is an immune-mediated endotheliopthy.^6^^,^^7^ One of the supporting means of this theory is the presence of anti-endothelial cell antibodies in serum of SS patients; however, the diagnostic value of this antibody is not yet clear, because it may be present in some of the other autoimmune disorders like systemic lupus erythematosus, rheumatoid arthritis, Sjogren syndrome and sarcoidosis.^18^

**Optical coherence tomography (OCT)**

One of the most important differential diagnoses of SS is MS; as will be discussed later, this is due to similarities in clinical features and MRI findings.^15^ In a new study, Brandt et al. compared the OCT and macular volume in patients with SS and patients with relapsing-remitting MS. OCT can quantify the thickness of retinal nerve fiber layer (RNFLT), and the macular volume represents the volume of the central retina. In their study, SS patients showed a characteristic pattern of patchy, and often severe, RNFLT thinning on RNFLT, and retinal damage on total macular volume. This is compatible with segmental involvement of retinal arterioles in SS. However in MS patients, the OCT showed diffuse thinning of RNFLT, which slightly enhanced on the temporal quadrant after the optic neuritis. They concluded that OCT is a useful diagnostic tool for differentiating SS from MS in challenging cases.^26^

**Diffusion tensor imaging (DTI)**

A new neuroimaging modality used for assessment of nerve fiber integrity of the white matter is DTI. DTI is a sensitive tool for detecting microstructural tissue damage, especially to nerve fibers, on the basis of fractional anisotropy. Fractional anisotropy reflects the spatial directionality of water diffusion that in microstructural damage of white matter decreases from normal values; this cannot be seen on conventional MRI. In patients with SS, reduced fractional anisotropy can be detected by DTI in the genu of the corpus callosum and the normal appearing prefrontal area on the conventional MRI. This disruption of fiber integrity in the normal appearing prefrontal area, which is detected by DTI, can explain the psychological manifestations of SS. Kleffner et al. evaluated nine patients with SS by DTI and concluded that a 25% reduction of fractional anisotropy in the corpus callosum of their patients, especially of the genu, is a characteristic finding for SS. They reported reduced fractional anisotropy in the genu of corpus callosum and prefrontal areas of the brain in patients with SS; but reduced fractional anisotropy in the body, rostrum and splenium of the corpus callosum in MS patients.^11^

**Differential Diagnosis**

There are several differential diagnoses for SS, and the most common are demyelinating processes like MS and ADEM; that is because of their autoimmune nature and similarities on MRI and clinical presentations; but hearing loss is a rare finding in MS, and retinal artery involvement in MS is peripheral retinal arteriolar vasculitis with cellular infiltration of the peripheral vitreous.^5^^,^^15^^,^^22^ There has been only one case of SS reported from Iran, who had first been diagnosed as MS because of her symptoms (paresthesia of four limbs) and MRI findings (multiple T2 and FLAIR high signal intensity lesions in periventricular areas); it was only after occurrence of hearing loss and tinnitus that the diagnosis of SS was suspected, and then confirmed by finding BRAO on ophthalmologic exam.^13^

There are some distinguishing features that help us to differentiate SS from MS and ADEM; (1) Corpus callosum involvement in SS is typically in the central portion of the corpus callosum and the periphery is spared, but in MS and ADEM the undersurface and septal interface of corpus callosum are involved. (2) Leptomeningeal enhancement is an uncommon finding in MS and ADEM but can be seen in up to 30% of patients with SS. (3) Involvement of deep grey matter including basal ganglia is a common finding in SS but unusual in MS. (4) On OCT, diffuse thinning of RNFLT is in favor of MS, but patchy and often severe thinning of RNFLT can be seen in SS patients.^10^^,^^18^^,^^24^^,^^25^

Other common differential diagnoses are infectious encephalitis, and both primary and secondary CNS vasculitis.^10^ One of these vasculitis syndromes is neuro-behçet, which can present as brainstem encephalitis, parenchymal cerebral disease (due to small vessel vasculitis with cortical and subcortical microinfarctions on MRI), and rarely with a corpus callosum involvement (as reported by Maaruf et al.).^27^^-^^29^

There is also wide variety of differential diagnoses considered in neurologic literature including cerebral autosomal dominant arteriopathy with subcortical infarcts and leukoencephalopathy (CADASIL), Creutzfeldt Jakob disease, stroke, malignant tumours, migraine with aura, psychosis, or myopathy, encephalopathy, lactate acidosis and stroke-like episodes (MELAS), and also Menière’s disease and Cogan syndrome in the otological field.^10^

**Treatment**

There is no general consensus on treatment of SS and, because of its rarity; there is no strong study to address this issue. Based on multiple case reports and case series, the best treatment may be immunosuppression with glucocorticoids; in those refractory to steroids, intravenous immunoglobulin (IVIG), plasma exchange, or more aggressive immunosuppression with azathioprine, cyclophosphamide, cyclosporine, methotrexate, mycophenolate mofetil, infliximab and rituximab can be used, based on severity of the disease and its clinical course.^4^^,^^10^^,^^15^^,^^18^ Of nine patients treated by plasma exchange in one study, six had symptomatic improvement, two had disease stabilization, and one was lost to follow-up.^8^ Many experts use antiplatelet agents (like aspirin and clopidogrel), and anti-vascular spasm agents (like nimodipine) as adjunctive therapy in patients with SS.^8^^,^^21^^,^^25^ Rennebohm et al. insist on early, aggressive, and sustained immunosuppression in encephalopathic forms of SS. Immunosuppression should be started as soon as possible and not be tapered very quickly.^17^ Kleffner et al. recommend use of IV methylprednisolone 1 g daily for 5 days, then 1 mg/kg daily oral prednisolone for 2-4 weeks, and then slow tapering (10-15% of the dose every 2 weeks) down to 10-15 mg/day, which should be continued for 6 months. Finally, slower tapering (2.5 mg every month) should be considered. They also used 80 mg/day of aspirin with their regimen.^10^ In severe cases, IVIG (0.4 g/kg/d for 5 days) can be used, and can be repeated monthly for 6 months.^4^^,^^5^^,^^22^ Another widely used immunosuppressive agent for SS is azathioprine with dosage of 2 mg/kg daily.^4^^,^^5^^,^^12^ Strong immunosuppression with IV cyclophosphamide, 1 g/day for 3 days and then 1 g monthly for 6 months, or with IV rituximab, 375 mg/m^2^ weekly for 4 weeks, have been used in very severe and refractory cases. There is no consensus on the duration of these immunosuppressive therapies, but it depends on the clinical course of the disease and patient’s condition.^4^^,^^5^^,^^12^^,^^30^

Deane et al. reported a 25-year-old pregnant woman with presentation of confusion, difficult walking, visual and hearing loss at 20 weeks of gestation, which diagnosed as SS and treated with aspirin, corticosteroids and IVIG with early improvement. However because of severe symptom recurrence during corticosteroid tapering at 33 weeks of gestation, they decided to early induction of delivery at 35 weeks of gestation and then treatment with IV cyclophosphamide and rituximab.^5^

**Course and Prognosis**

SS can be a self-limiting disease; it can improve, without any treatment, during the course of 1-2 years, but some sequels and relapses may happen. Some patients will develop epilepsy, dementia, and permanent vision or hearing loss as squela.^8^ therefore, early diagnosis and treatment is important to reduce the sequels.^4^^,^^6^^,^^15^^,^^21^ The sequels of encephalopathy occur in 60-70% of patients, but are mild in the majority of cases.^9^ In contrast to encephalopathy and vision loss that usually remit with no or mild sequels, SNHL in SS is severe and most often irreversible, and the patient may need hearing aid devices; cochlear implants may help in severe cases. Due to the risk of relapses, even decades after the first presentation, life-long monitoring of these patients is recommended.^10^

Susac in a review of his patients described three different courses of the disease; first is a monocyclic disease, characterized by some fluctuations, but eventually remitting during a course of 1-2 years; the second is polycyclic disease with remission between episodes that lasts more than 2 years; and the third is a chronic continuous form without remission for more than 2 years.^7^

## Notes

**How to cite this article: **Nazari F, Azimi A, Abdi S. What is Susac syndrome? - A brief review of articles. Iran J Neurol 2014; 13(4): 209-14.
